# Individualized luteal phase support in frozen-thawed embryo transfer after intramuscular progesterone administration might rectify live birth rate

**DOI:** 10.3389/fendo.2024.1412185

**Published:** 2024-06-28

**Authors:** Fazilet Kübra Boynukalin, Yusuf Aytac Tohma, Zalihe Yarkıner, Meral Gultomruk, Gürkan Bozdag, Sinan Ozkavukcu, Mustafa Bahçeci, Berfu Demir

**Affiliations:** ^1^ Infertility Department, Bahçeci Fulya IVF Center, Istanbul, Türkiye; ^2^ Department of Obstetrics and Gynecology, Faculty of Medicine, Üsküdar University, Istanbul, Türkiye; ^3^ Infertility Department, Bahçeci Ankara IVF Center, Ankara, Türkiye; ^4^ Department of Obstetrics and Gynecology, Faculty of Medicine, Atılım University, Ankara, Türkiye; ^5^ Department of Basic Sciences and Humanities, Faculty of Arts and Sciences, Cyprus International University, Nicosia, Cyprus

**Keywords:** hormone replacement therapy, frozen embryo transfer, intramuscular progesterone, luteal phase support, rescue progesterone

## Abstract

**Background:**

The serum P concentrations are suggested to have an impact on pregnancy outcome. However there is no consensus about the optimal progesterone cut-off during the luteal phase. Few studies evaluated the effectiveness of a “rescue protocol” for low serum P concentrations and most of these studies used vaginal progesterone administration. There is paucity of data on the effectiveness of rescue protocol using intramuscular progesterone (IM-P) in frozen-thawed embryo transfer (FET).

**Methods:**

This study is a retrospective cohort study included 637 single or double blastocyst FETs with artificially prepared endometrium receiving 100 mg IM progesterone (P) after incremental estrogen treatment. Serum P concentrations were evaluated using blood samples obtained 117-119 hours after the first IM-P administration and 21 ± 2 hours after the last IM-P administration. Patients with serum P concentrations <20.6 ng/ml on the ET day were administrated 400 mg vaginal progesterone for rescue.

**Results:**

Demographic and cycle characteristics were similar between patients receiving rescue vaginal P (embryo transfer (ET)-day P concentration < 20.6 ng/ml) and patients who did not need rescue vaginal P (ET-day P concentration ≥ 20.6 ng/ml). Clinical pregnancy, miscarriage, and live birth rates were similar between two groups: 52.9%(45/85) vs 59.6%(326/552), p=0.287; 11.1%(5/45) vs 14.1%(46/326), p=0.583; and 47.1%(40/85) vs 50.7%(280/552), p=0.526, respectively. Logistic regression analysis revealed that the female age (p = 0.008, OR=0.942, 95% CI = 0.902–0.984) and embryo quality (ref: good quality for moderate: p=0.02, OR=0.469, 95% CI =0.269–0.760; for poor: p=0.013, OR= 0.269, 95% CI = 0.092–0.757) were independent variables for live birth. Following rescue protocol implementation, ET-day P concentration was not a significant predictor of live birth.

**Conclusions:**

Rescue vaginal P administration for low ET day serum P concentrations following IM-P yields comparable live birth rates.

## Introduction

In recent years, increasing attention has been paid to the individualization of ovarian stimulation (OS) and luteal phase support (LPS) in all fields of medical treatment. It is crucial to maximize efficacy and safety and to minimize the treatment burden, side effects, and cost. At the same time, a paradigm shift has occurred from fresh embryo transfers (ETs) to frozen embryo transfers (FETs) in IVF treatments, and the individualization of LPS in FET cycles has become the center of attention. There is no consensus on which endometrial preparation protocol is superior in FET (1, 2). The endometrial preparation protocol should be considered when regarding the contribution of LPS to FET.

LPS is essential for artificial cycle (AC) endometrial preparation protocols as no functional corpus luteum is present. Progesterone (P) is crucial for transforming a receptive endometrium and the maintenance of a pregnancy. P administration routes, dosages, and timing, as well as serum P concentrations, have been the focus of many studies. LPS without luteal P monitoring was once standard practice. However, recent studies have reported interpersonal variations that might affect pregnancy outcomes (3). The majority of these studies involved vaginal progesterone (V-P), which is commonly used in Europe (4–10). Although there is no consensus on the optimal cut-off value during the luteal phase, P levels lower than 10 ng/ml around ET are insufficient for optimal pregnancy outcomes after V-P administration. Only a few studies (11, 12) have evaluated the factors that might affect serum P concentrations after V-P administration.

There is scarce of data for individualized LPS after intramuscular progesterone (IM-P) administration. V-P and IM-P administration routes have different local and systemic P concentration patterns. Hence, the results of the studies on V-P administration cannot be applied to IM-P administration. The effect of serum P concentrations on pregnancy outcomes has also been evaluated following IM-P administration in AC-FET (13–16). Two of these studies were prospective (13, 14). Our previous study revealed that, as already published regarding V-P, there is a minimum threshold of serum ET day P concentrations for optimal pregnancy outcomes for IM-P (14).

Although the measurement of serum P concentration seems simple, it is the only available parameter for the individualization of LPS after AC-FET. In parallel with the “one size does not fit all” concept. Following the results of our first study (14), we activated individualized LPS after AC-FET using IM-P. Rescue V-P was administered if the patient’s serum P concentration was < 20.6 ng/ml on the ET day in cases of frozen-thawed blastocyst transfers. This retrospective study aimed to evaluate if individualized LPS improved pregnancy outcomes in cases with a low serum P concentration. Additionally, the factors that are predictive for patients at risk for low serum P concentrations on the ET day were evaluated, which might facilitate the optimization of LPS.

## Materials and methods

### Ethical approval

This study was approved by the Bahçeci Ethical Committee on 22 September 2022 (reference number 106–2022).

### Study design

A retrospective cohort study of 637 single or double blastocyst FET cycles was performed at Bahçeci Ankara IVF Center between November 2019 and February 2022.

### Study population

**Figure 1:**
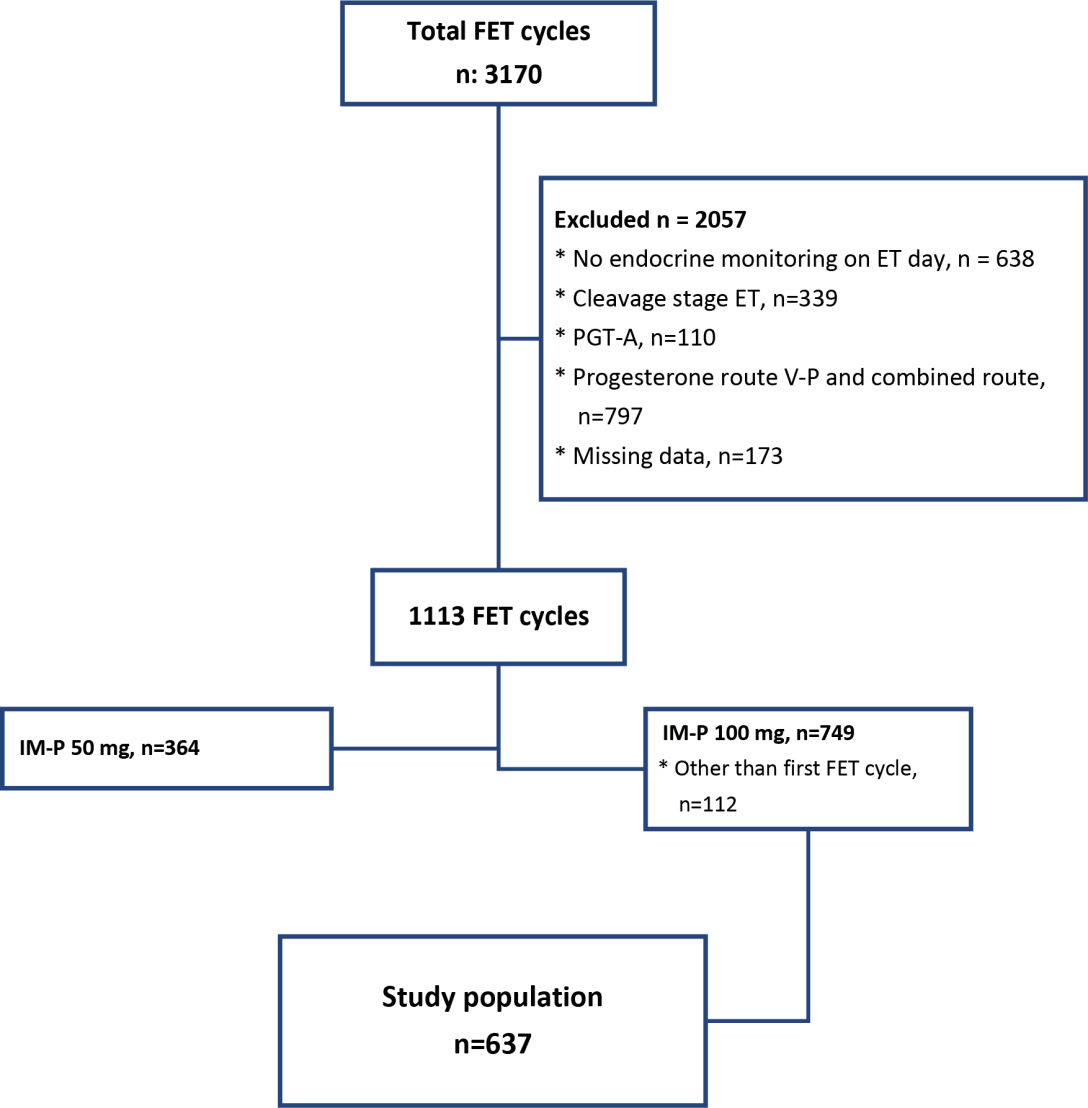
Flow-chart.

During the study period, a total of 3170 FETs were performed (**Figure 1**). The study included 637 patients’ FET cycles. Inclusion criteria were AC, one or two blastocyst transfer FET cycles, in 20–46-year-old women, whose serum P concentrations were monitored after 100 mg IM-P administration. Exclusion criteria were cleavage ET, PGT-A cycles, natural or stimulated endometrial preparation, AC cycles with IM-P doses other than 100 mg, AC cycles with V-P administration, or AC cycles with combined P administration routes. Cycles with missing data were secondarily excluded. Patients were included only once in the analysis.

### Clinical and laboratory procedures

OS, oocyte retrieval, denudation, intracytoplasmic sperm injection (ICSI), embryo culture, vitrification, and warming procedures were performed as previously described (14). The Gardner and Schoolcraft Classification System was used for blastocyst morphology evaluation (17). The blastocysts were categorized as follows: good (3AA, 3AB, 3BA, 4AA, 4AB, 4BA, 5AA, 5AB, and 5BA.), moderate (3BB, 3BC, 4BB, 4BC, 5BB and 5BC);, and poor (3CB, 3CC, 4CB, 4CC, 5CB, 5CC).

Endometrial preparation for AC-FET was initiated on the second or third day of the menstrual cycle, as was oral estrogen (Estrofem, Novo Nordisk, Istanbul, Turkey) in an incremental protocol: 4 mg/day on days 1–4, 6 mg/day on days 5–8, and 8 mg/day on days 9–12. Transvaginal ultrasonography (TV-USG) was performed between the 10^th^ and 13^th^ day; if the endometrial thickness was > 7 mm and the serum P concentration was < 1.5 ng/ml, 100 mg of IM-P (Progestan, Koçak Farma, Turkey) was administered. Estradiol (E_2_) levels before P administration were not considered as a criterion before P administration. There is no agreement in the literature on the optimal dosage of progesterone for luteal phase support. The typical IM P dose ranges from 25 to 100 mg/day (18). In FET cycles this range is from 50 to 100 mg. Little information comparing the pregnancy outcomes of regarding IM-P dosage in planned FET cycles has been reported. In a common policy in our clinic 100 mg IM-P is used (19).

The first dose of IM-P was injected at 4:00 PM, and subsequent doses were repeated every 24 hours at the same time. ET was performed on the sixth day of P administration between 3:00 PM and 5:00 PM under ultrasonographic guidance; a blood sample to measure ET-day P concentrations was collected between 1:00 and 3:00 PM. Patients with a serum P < 20.6 ng/ml—the threshold reported in a previous study—were given an additional 400 mg of V-P (Progestan, Koçak Farma, Turkey) daily apart from the standard 100 mg of IM-P in the evening after ET as individualized LPS. Patients with ET-day P concentrations ≥ 20.6 ng/ml were continued on the standard 100 mg of IM-P. LPS was performed until the 10^th^ week of pregnancy or until the day of pregnancy if negative in both groups.

A GnRH agonist usage was depended on the clinician’s preference and a single dose of 3,75 mg leuprolide acetate (Lucrin Depot, AbbVie, Japan) was administered on the 20^th^ day of the preceding cycle and estrogen was started on the 2^nd^ or 3^rd^ day of menstruation as described above.

Blood samples were evaluated by an electrochemiluminescence immunoassay (CobasV ^®^ Elecsys Progesterone III, Roche Diagnostics GmbH, Germany) with a measured sensitivity and total imprecision of 0.03 mg/l and < 7%, respectively.

### Outcome measures

Clinical pregnancy rate (CPR) was defined as the detection of an intrauterine gestational sac via TV-USG per ET, and live birth rate (LBR) was defined as the number of deliveries beyond 24 weeks of pregnancy per ET. Miscarriage was defined as the loss of clinical pregnancy before gestational week 12.

### Statistics

This study included a total sample size of 637 patients with single blastocyst FET cycles. The study accounted for patient demographics as well as cycle characteristics. Descriptive information was recorded for exploratory data analysis. As the sample size was sufficiently large, continuous variables following a normal distribution were evaluated using the Kolmogorov–Smirnov test. Categorical variables were compared between Groups 1 and 2 with the chi-squared test. To determine which factors affected the outcome of an ongoing pregnancy, a binary logistic regression analysis was performed with a forward stepwise conditional procedure.

The association between variables (patient demographics and cycle characteristics) and P concentrations on the ET day were analyzed using Pearson’s correlation coefficient test. The effect of each variable on P concentration on the ET day was analyzed separately, using each variable as an independent factor and P concentration on the ET day as a dependent variable, in the univariate linear regression model. Based on the correlation analysis and univariate linear regression results, multivariate linear regression was carried out to determine the effects of all significant variables on P concentration on the ET day simultaneously. A p-value < 0.05 was considered statistically significant, and regarding the multivariate model, only the significant factors are reported in the results. Statistical analyses were performed with SPSS Statistics for Windows, Version 25 (IBM Corp., Armonk, NY, USA).

## Results

### Basic characteristics

This study included a total of 637 patients’ first AC-FET cycles. Patient demographics and cycle parameters are presented in **Table 1**. The mean overall female age was 31.9 ± 5.1 years, and the mean body mass index (BMI) was 26.72 ± 4.68 kg/m^2^. The mean serum P, estradiol (E_2_), and luteinizing hormone (LH) concentrations on the blastocyst ET day were 30.20 ± 9.28 ng/ml, 266.07 ± 90.89 pg/ml, and 3.48 ± 3.66 IU, respectively. The CPR, miscarriage rate, and LBR were 58.2% (371/637), 13.7% (51/371), and 50.2% (320/637), respectively. In 13.3% of the patients (85/637), serum P concentration was lower than 20.6 ng/ml.

**Table 1:** Patient demographics and cycle characteristics.

Female age (years)	
*Mean ± SD*	**31.89 ± 5.101**
BMI (kg/m^2^)	
*Mean ± SD*	**26.72 ± 4.68**
Type of infertility	
*Primary, n (%)*	**442/637 (69.4)**
Duration of infertility (years)	
*Mean ± SD*	**4.72 ± 4.005**
Causes of infertility	
*Male factor, n (%)*	**217/637 (34.1)**
*DOR, n (%)*	**81/637 (12.7)**
*PCOS, n (%)*	**105/637 (16.5)**
*Tubal factor, n (%)*	**31/637 (4.9)**
*Endometriosis, n (%)*	**46/637 (7.2)**
*Unexplained, n (%)*	**147/637 (23.1)**
*Uterine factor, n (%)*	**10/637 (1.6)**
E_2_ level on estrogen administration day (pg/ml)	
*Mean ± SD*	**34.17 ± 20.61**
P level on estrogen administration day (ng/ml)	
*Mean ± SD*	**0.31 ± 0.21**
LH level on estrogen administration day (IU/L)	
*Mean ± SD*	**4.88 ± 3.92**
E_2_ level on progesterone administration day (pg/ml)	
*Mean ± SD*	**276.24 ± 135.63**
*Median (Maximum–Minimum)*	**261.80 (1280–0)**
P level on progesterone administration day (ng/ml)	
*Mean ± SD*	**0.20 ± 0.21**
LH level on progesterone administration day (IU/L)	
*Mean ± SD*	**10.17 ± 9.40**
Endometrial thickness (mm)	
*Mean ± SD*	**10.66 ± 1.77**
Duration of estrogen administration until IM-P usage (days)	
*Mean ± SD*	**13.86 ± 1.26**
GnRH agonist usage	
*n (%)*	**139/637 (21.8)**
ET-day E_2_ level (pg/ml)	
*Mean ± SD*	**266.07 ± 90.89**
ET-day P level (ng/ml)	
*Mean ± SD*	**30.20 ± 9.28**
ET-day LH level (ng/ml)	
*Mean ± SD*	**3.48 ± 3.66**

### Patients with serum P concentrations < 20.6 ng/ml and individualized LPS vs patients with P concentrations ≥ 20.6 ng/ml

Patient characteristics and cycle parameters are presented in **Table 2**. Patients with serum P concentrations < 20.6 ng/ml and individualized LPS had a significantly higher BMI (27.6 ± 5.19 kg/m^2^ vs 26.81 ± 4.36 kg/m^2^, p = 0.047). The other cycle and demographic parameters were similar between the two groups. No differences were observed in the CPR, miscarriage rate, and LBR between the two groups. The CPR was 52.9% (45/85) vs 59.6% (326/552), p = 0.287; the miscarriage rate was 11.1% (5/45) vs 14.1% (46/326), p = 0.583; and the LBR was 47.1% (40/85) vs 50.7% (280/552), p = 0.526 in patients with serum P concentrations < 20.6 ng/ml and individualized LPS vs patients with serum P concentrations ≥ 20.6 ng/ml, respectively.

**Table 2:** Comparison of patients with serum P < 20.6 ng/ml and individualized LPS and patients with serum P ≥ 20.6 ng/ml.

	ET-day P concentration < 20.6 ng/ml	ET-day P concentration ≥ 20.6 ng/ml	p
**Female age**	30.84 ± 4.52	31.44 ± 5.02	0.33
**BMI**	27.6 ± 6.19	26.41 ± 4.36	0.047
**Duration of infertility**	3.99 ± 2.97	4.74 ± 4.14	0.393
Causes of infertility			
**Male factor**	32/85 (37.6%)	185/552 (33.5%)	0.33
**DOR**	7/85 (8.2%)	74/552 (13.4%)	
**PCOS**	16/85 (18.8%)	89/552 (16.1%)	
**Tubal factor**	6/85 (7%)	25/552 (4.5%)	
**Endometriosis**	7/85 (8.2%)	39/552 (7.1%)	
**Unexplained**	16/85 (18.8%)	131/552 (23.7%)	
**Uterine factor**	1/85 (1.2%)	9/552 (1.6%)	
**Endometrial thickness**	10.48 ± 1.99	10.66 ± 1.72	0.277
**E_2_ level on P administration day**	282.24 ± 154.98	277.24 ± 130.07	0.787
**P concentration on P administration day**	0.21 ± 0.27	0.20 ± 0.18	0.647
**Duration of estrogen administration until P administration**	13.78 ± 2.32	13.56 ± 1.33	0.821
GnRH agonist			
**Yes**	20/85 (23.5%)	119/552 (21.6%)	0.512
**No**	65/85 (86.5%)	433/552 (78.4%)	
**No of embryos transferred**	1.1 ± 0.26	1.2 ± 0.39	0.09
Embryo quality			
**Poor**	3/91(3.3%)	30/654(4.6%)	
**Moderate**	55/91(60.4%)	375/654(57.3%)	0.777
**Good**	33/91(36.3%)	249/654(38.1%)	
**Clinical pregnancy rate**	45/85 (52.9%)	326/552 (59.6%)	0.287
**Live birth rate**	40/85 (47.1%)	280/552 (50.7%)	0.526
**Miscarriage rate**	5/45 (11.1%)	46/326 (14.1%)	0.583

The logistic regression analysis for LBR after adjusting for possible confounders, such as the female age, BMI, embryo quality, number of embryos transferred, infertility duration and causes of infertility, and ET-day serum P concentration and endometrial thickness, showed that the ET-day serum P concentration did not affect the live birth. Female age (p = 0.008, OR = 0.942, 95% CI = 0.902–0.984) and embryo quality (ref: good quality, for moderate quality: p = 0.02, OR = 0.469, 95% CI = 0.269–0.760; for poor quality: p = 0.13, OR = 0.269, 95% CI = 0.092–0.757) were independent factors that affected the LBR (**Table 3**).

**Table 3:** Multivariate logistic regression analysis for live birth.

	B	S.E.	Wald	df	Sig.	OR	95% C.I.for EXP(B)	
							Lower	Upper
**Female age**	-.060	.022	7.095	1	.008	.942	.902	.984
**Embryo quality** **(Ref: Good)**			14.210	2	.001			
**Moderate**	-.757	.246	9.439	1	.002	.469	.289	.760
**Poor**	-1.333	.538	6.140	1	.013	.264	.092	.757
**Constant**	1.720	1.443	1.422	1	.233	5.587		

*Variable(s) entered on: Female age, BMI, embryo quality, number of embryos transferred, infertility duration and causes of infertility, ET day serum P concentration and endometrial thickness.

### Factors that affect serum P concentrations on the day before FET

The correlation of patient and cycle characteristics with serum P concentrations on the ET day was evaluated using Pearson’s correlation test. The ET-day serum P concentration was correlated with BMI (R = –0.12, p = 0.002) and ET-day E_2_ level on P administration day (R = 0.135, p = 0.001) but not with the female age (R = 0.074, p = 0.063); **Supplementary Table 1**. Similarly, the univariate analysis showed that serum P levels on the ET day were statistically significantly affected by BMI (p = 0.005) and ET-day E_2_ levels (p = 0.01) separately (**Supplementary Table 2**). When multivariate linear regression was performed to correct for potential confounders, a significant negative correlation of ET-day P concentrations with BMI (β = –0.264, 95% CI = –0.417 to –0.11, p = 0.001) was observed. In contrast to this finding, a significant positive correlation was seen between the P concentration and ET-day E_2_ level (β = 0.01, 95% CI = 0.003–0.018, p = 0.009) (**Supplementary Table 3**).

## Discussion

This study evaluated individualized LPS in cases with low serum P concentrations on the ET day after IM-P administration following blastocyst-stage FET. The study showed that individualized LPS using rescue V-P administration in addition to IM-P in cases with an ET-day P concentration < 20.6 ng/ml can result in a similar LBR in cases with ET day P concentration > 20.6 ng/ml without rescue P administration. Moreover, after the individualization of LPS, the ET-day serum P concentration did not significantly affect live birth.

Since corpus luteum function is absent, and due to exogenous E_2_ administration, exogenous P administration is crucial in AC-FET cycles. P administration is usually initiated 10–14 days after E_2_ administration. There is no definite consensus on the best route, dosage, or length of exposure to P in AC-FET cycles. A recent randomized control study compared vaginal, IM and oral progesterone routes in AC-FET cycles and reported similar live birth rates but it is with a higher side effects in IM arm (20).

Systemic P levels in AC-FET cycles are a topic of interest in determining the most efficient and effective P administration method. The optimal cut-off level of systemic P for a successful AC-FET cycle differs between administration routes. There is no doubt that serum P concentrations fluctuate after corpus luteum formation and after V-P, IM-P, and subcutaneous P administration (21). The half-life of IM-P is significantly longer than those of subcutaneous P and V-P. Steady serum P concentrations have been achieved 48 hours after IM-P administration (22). The local P concentration is significantly higher than the systemic P concentration after V-P administration (23). Even though the difference between the local and systemic P concentrations is not as apparent for IM-P administration as for V-P administration, the systemic P concentration after IM-P administration does not completely represent local concentrations (24). Based on reports of the serum P concentration on the day of ET or the previous day after V-P administration in AC-FET, low serum P concentrations are generally considered to negatively impact pregnancy outcomes (6–9). However, each study reported different cut-off levels and a recent meta-analysis reported high interstudy heterogeneity (3). Although there is no consensus on serum P concentrations, the usage of rescue protocols was evaluated in a few studies and found to eliminate the negative effect of low serum P concentrations after V-P administration (25–27).

In a recent metanalysis, relative risk for low P concentrations on ET day for V-P administration was 1.3 (3). To our knowledge, there is no randomized control study evaluating the effect of rescue dosage for V-P or IM-P administration. Our initial study reported a correlation between serum P levels and pregnancy outcomes, warranting an intervention in patients with low P levels (14). Based on the results of that study, we used the cut-off level of 20.6 ng/ml on the ET day. This cut off level was obtained from ROC curve analysis with moderate accuracy. We sought to increase P supplementation in women with low P levels as a straightforward solution to this problem. and administered the rescue protocol using a different route (400 mg V-P administration starting the evening of the ET day). Additionally, our study retrospectively evaluated the effect of rescue dosage on LBR, and the results reinforce this strategy, with the rescue protocol seeming to normalize the pregnancy outcome.

A rescue protocol after IM-P administration was only evaluated in an oocyte donation cycle study (15). However, in this study, the IM-P dosage was not standard (between 50 and 100 mg), serum P concentration was evaluated on the cleavage-stage ET day, and if P < 20 ng/ml, the dose of IM-P was increased by 50–100%. Nevertheless, the rescue dosage LBR was lower than that of patients with serum P > 20 ng/ml. In this study, donor cycles were performed on patients with 1, 2, or 3 embryos transferred on day 3. The timing of starting administration and measuring P levels, as well as the interval of ET, were not reported. The number and quality of embryos transferred were also not reported for the comparison groups, which might cause a bias. In contrast to this study, a recent retrospective study reported that increasing IM-P dosage from 50 mg to 75 mg nightly if P levels were below 15 ng/mL gave similar results to those seen for patients with P levels greater than 15 ng/ml (28). The determination of a cut-off value was not explained in this study. The study included 903 patients, 58% of whom needed a rescue dose, which can be interpreted as most patients in this study not being given an optimal P dose before ET.

Recent evidence suggests that even when the same route and dosage of P is administered, significant interpersonal variations occur. These variations are reported to affect pregnancy outcomes with an area under the curve of 0.72 (14). To improve pregnancy outcomes in AC-FET cycles, two strategies can be followed: (i) individualization of LPS and (ii) optimization of LPS. Currently, monitoring serum P concentrations before FET is the only tool for the individualization of LPS. Serum P is monitored, and rescue P is administered in case of suboptimal P levels. The optimization of LPS can be achieved by dose adjustment according to the factors that affect serum P concentrations.

There is a paucity of data on the factors affecting serum P concentrations, and the studies that have been performed reported the results of V-P administration. Gonzalez-Foruria et al. (11) evaluated the clinical factors related to serum P concentration using 685 single cryopreserved blastocyst transfers under AC-FET with V-P. Body weight, age, time of blood sampling, and a history of low P were associated with P concentrations before blastocyst AC-FET. In a recent study, 915 single blastocyst FET transfers were evaluated; parity, BMI, and ethnicity were found to be associated with serum P concentrations on the day of the ET in AC-FET cycles with V-P administration (12). Our data revealed that, even after the utilization of same dosage of IM-P, still 13.3% of the patients had lower serum concentration than 20.6ng/mL. We noticed that P concentrations on the day of ET were negatively correlated to BMI but positively correlated with E2 concentrations on the day of ET. However, no correlation with female age, etiology of infertility, E2, P and LH concentrations on the day of P commencement or duration of estrogen treatment were found. Therefore, not only the patient or cycle characteristics but also pharmacokinetic and pharmacogenetic parameters may affect the serum P values.

Our study revealed that increased BMI lowers serum P concentrations. As BMI is known to affect drug distribution, metabolism, and excretion, it is possible that increasing BMI alters the pharmacokinetics of IM-P (29, 30). Some studies have reported poorer outcomes with high BMI in FET cycles (31–34), and impaired outcomes are also suggested to be related to endometrial receptivity (35). However, it can be hypothesized that a low serum P concentration is more frequently seen in patients with a high BMI, which may be the underlying reason for impaired endometrial receptivity.

Another finding of our study was the positive correlation between ET-day E_2_ levels and ET-day P concentrations. The response to estrogen supplementation depends on pharmacokinetic parameters. Genetic alterations among patients may influence ET-day E_2_ levels, and the same mechanism may also affect the response to IM-P administration. The positive correlation between ET-day E_2_ levels and ET-day P concentrations may indicate interpatient and intra-patient pharmacogenetic markers. In a recent study, effect of E_2_ levels on FET was evaluated and showed no correlation between E2 levels and P4 levels (36). The estrogen treatment protocol used was 6 mg once midnight oral administration. In our study incremental estrogen treatment was used and the dosage was increased up to 8 mg.

GnRH agonist can be used to suppress ovulation which may cause a cycle cancellation and it may stabilize the hormonal profile of AC-FET cycle. The recent meta-analysis revealed that it is uncertain if using GnRH agonist to suppress the ovulation improves clinical outcome (1). Although one may suggest that changes in hormonal profile during AC-FET cycle may cause a difference in ET day P concentrations, we did not find any evidence supporting this assumption in our data. The serum P concentrations on day of ET did not affect pregnancy outcome irrespective from the preference of GnRH agonist suppression.

Further RCTs are warranted to evaluate the most effective rescue type, route, and dosage in AC-FET after IM-P administration for the individualization of LPS. According to our findings, dose adjustment may lead to the establishment of the best protocols for the optimization of LPS. Despite the long half-life and steady serum P concentrations, it would be pretentious to claim that it will reflect the endometrial concentration and its effect. Detailed research is needed to understand the biological mechanisms influencing serum P concentration, including pharmacokinetic and pharmacogenetic parameters. Not only the serum P concentrations but also the endometrial microenvironment should be evaluated using tissue samples, metabolomics, or transcriptomics to model the best implantation process in systems medicine.

The strengths of this study are as follows: (i) strict inclusion criteria were used in terms of dose (100 mg) and rhythm (administration at 4:00 PM); (ii) serum P concentrations were analyzed by the same immunoassay and within a specific time range (just before the ET-day administration, in the afternoon at 3:00 PM) on the ET day; (iii) a significant number of demographic and endocrinological parameters were evaluated; and (iv): regression analysis for the main outcome was performed. The main limitations of this study include (i) its retrospective design, which can lead to a risk of selection bias; (ii) single serum P measurement; and (iii) a lack of information about perinatal outcomes.

In conclusion, this study verifies that the administration of V-P confirms pregnancy outcomes in patients with low P levels.

## Data availability statement

The original contributions presented in the study are publicly available. This data can be found here: dx.doi.org/10.6084/m9.figshare.24769785

## Ethics statement

The studies involving humans were approved by T. C. Bahçeci Sağlik Grubu Etik Kurul Onayi. The studies were conducted in accordance with the local legislation and institutional requirements. Written informed consent for participation in this study was provided by the participants’ legal guardians/next of kin.

## Author contributions

KB: Conceptualization, Data curation, Formal analysis, Investigation, Methodology, Resources, Software, Supervision, Validation, Visualization, Writing – original draft, Writing – review & editing. YT: Conceptualization, Data curation, Formal analysis, Investigation, Methodology, Resources, Software, Supervision, Validation, Visualization, Writing – review & editing. ZY: Data curation, Formal analysis, Methodology, Project administration, Supervision, Validation, Writing – review & editing. MG: Conceptualization, Data curation, Formal analysis, Funding acquisition, Investigation, Methodology, Project administration, Resources, Software, Supervision, Validation, Visualization, Writing – review & editing. GB: Conceptualization, Data curation, Formal analysis, Investigation, Methodology, Supervision, Validation, Writing – review & editing. SO: Data curation, Formal analysis, Methodology, Supervision, Validation, Writing – review & editing. MB: Writing – review & editing. BD: Conceptualization, Formal analysis, Writing – review & editing.

## Supplementary material

Supplementary Table 1 Correlation analysis of patient and cycle characteristics with serum P concentration on ET day.

Supplementary Table 2 Univariate analysis for factors associated with serum P values on blastocyst ET day.

Supplementary Table 3 Multivariate linear regression factors associated with serum P concentrations on the day of embryo transfer after IM-P. **Linear Regression Model, Dependent variable: ET-day P. Independent variables used in Model:** Female age, BMI, ET-day E_2_, Type of Infertility, P level on estrogen administration day (ng/ml), LH level on estrogen administration day (IU/L), E_2_ level on progesterone administration day (pg/ml), Duration of estrogen administration until IM-P usage (days), P level on progesterone administration day (ng/ml), LH level on progesterone administration day (IU/L), infertility diagnosis.
