# Supplementary material for: Individualized luteal phase support in frozen-thawed embryo transfer after intramuscular progesterone administration might rectify live birth rate
Source: Front Endocrinol (Lausanne). 2024 Jun 28;15:1412185. doi: 10.3389/fendo.2024.1412185 (PMC11239543; doi:10.3389/fendo.2024.1412185)
Supplement: Supplementary Table 1 — Correlation analysis of patient and cycle characteristics with serum P concentration on ET day. [file Table_1.docx]

**Supplementary Table 1:** Correlation analysis of patient and cycle characteristics with serum P concentration on ET day.

| Parameter | R | p |
| --- | --- | --- |
| Female age (years) | 0.074 | 0.063 |
| BMI (kg/m2) | -0.120 | 0.002 |
| Infertility type |  |  |
| *Primary* | -0.057 | 0.154 |
| *Secondary* | 0.057 | 0.154 |
| Causes of infertility |  |  |
| *Male factor (n/%)* | -0.068 | 0.089 |
| *DOR (n/%)* | 0.047 | 0.238 |
| *PCOS (n/%)* | 0.012 | 0.760 |
| *Tubal Factor (n/%)* | 0.017 | 0.675 |
| *Anovulatuar (n/%)* | -0.055 | 0.166 |
| *Endometriosis (n/%)* | -0.021 | 0.593 |
| *Unexplained (n/%)* | 0.043 | 0.281 |
| *Uterine factor (n/%)* | -0.003 | 0.931 |
| E_2_ Level on estrogen administration day (pg/ml) | 0.0001 | 0.997 |
| P Level on estrogen administration day (ng/ml) | 0.031 | 0.564 |
| LH Level on estrogen administration day (IU/L) | -0.001 | 0.979 |
| Duration of estrogen administration until IM-P usage (days) | -0.013 | 0.745 |
| E_2_ Level on progesterone administration day (pg/ml) | -0.007 | 0.852 |
| P Level on progesterone administration day (ng/ml) | 0.022 | 0.583 |
| LH Level on progesterone administration day (IU/L) | -0.058 | 0.146 |
| ET-day E_2_ Level (pg/ml) | 0.135 | 0.001 |
| GnRH agonist |  |  |
| Yes (n/%) | -0.016 | 0.686 |
| No (n/%) | 0.016 | 0.686 |

**Supplementary Table 2:** Univariate analysis for factors associated with serum P values on blastocyst ET day.

| **Model Term** | **Coefficient** | **Standard Error** | **t-test value** | **p-value** | **95% Confidence Interval** | |
| --- | --- | --- | --- | --- | --- | --- |
|  |  |  |  |  | **Lower** | **Upper** |
| **Female age (years)** | 0.134 | 0.072 | 1.866 | 0.063 | -0.007 | 0.276 |
| **BMI (kg/m2)** | -0.227 | 0.081 | -2.806 | 0.005 | -0.386 | -0.068 |
| **E_2_ level on estrogen administration day (pg/ml)** | 0.016 | 0.024 | 0.659 | 0.51 | -0.031 | 0.062 |
| **Type of infertility** |  |  |  |  |  |  |
| **Primary** | -1.172 | 0.821 | -1.428 | 0.154 | -2.784 | 0.439 |
| **Secondary** | 1.172 | 0.821 | 1.428 | 0.154 | -0.439 | 2.784 |
| **P level on estrogen administration day (ng/ml)** | 1.424 | 2.3 | 0.619 | 0.536 | -3.099 | 5.947 |
| **LH level on estrogen administration day (IU/L)** | -0.005 | 0.124 | -0.039 | 0.969 | -0.248 | 0.239 |
| **E_2_ level on progesterone administration day (pg/ml)** | -0.132 | 0.299 | -0.441 | 0.659 | -0.719 | 0.455 |
| **Duration of estrogen administration until IM-P usage (days)** | -0.013 | 0.712 | -0.132 | 0.769 | -0.081 | 2.12 |
| **E_2_ level on progesterone administration day (pg/ml)** | -0.001 | 0.003 | -0.186 | 0.852 | -0.006 | 0.005 |
| **P level on progesterone administration day (ng/ml)** | -1.419 | 1.783 | -0.796 | 0.426 | -4.919 | 2.082 |
| **LH level on progesterone administration day (IU/L)** | -0.029 | 0.039 | -0.736 | 0.462 | -0.106 | 0.048 |
| **ET-day E_2_ Level (pg/ml)** | 0.01 | 0.004 | 2.6 | 0.01 | 0.003 | 0.018 |
| **Endometrial thickness (mm)** | -0.174 | 0.213 | -0.814 | 0.416 | -0.592 | 0.245 |
| **Diagnosis** |  |  |  |  |  |  |
| **Male Factor** | -1.321 | 0.775 | -1.705 | 0.089 | -2.842 | 0.201 |
| **Female Factor + Unexplained** | - | - | - | - | - | - |
| **GnRH agonist** |  |  |  |  |  |  |
| **Yes** | -0.37 | 0.914 | -0.405 | 0.686 | -2.165 | 1.425 |
| **No** | - | - | - | - | - | - |

**Supplementary Table 3:** Multivariate linear regression factors associated with serum P concentrations on the day of embryo transfer after IM-P.

| **Model Term** | **Coefficient (ß)** | **Standard Error** | **t-test value** | **p-value** | **95% Confidence Interval** | |
| --- | --- | --- | --- | --- | --- | --- |
|  |  |  |  |  | **Lower** | **Upper** |
| **Intercept** | 34.142 | 2.378 | 14.356 | <0.0001 | 29.472 | 38.813 |
| **BMI (kg/m2)** | -0.264 | 0.078 | -3.365 | 0.001 | -0.417 | -0.110 |
| **ET-day E_2_ Level (pg/ml)** | 0.01 | 0.004 | 2.607 | 0.009 | 0.003 | 0.018 |

**Linear Regression Model, Dependent variable: ET-day P**

**Independent variables used in Model:** Female age, BMI, ET-day E_2_, Type of Infertility, P level on estrogen administration day (ng/ml), LH level on estrogen administration day (IU/L), E_2_ level on progesterone administration day (pg/ml), Duration of estrogen administration until IM-P usage (days), P level on progesterone administration day (ng/ml), LH level on progesterone administration day (IU/L), infertility diagnosis
